# Supplementary material for: Hormonal contraceptives and body composition by use of stable isotope dilution techniques among women of reproductive age in Nyeri County, Kenya
Source: PLoS One. 2025 Sep 12;20(9):e0332299. doi: 10.1371/journal.pone.0332299 (PMC12431291; doi:10.1371/journal.pone.0332299)
Supplement: Attachment — Submitted filename: Hormonal Contraceptive_Maria.pdf [file pone.0332299.s001.pdf]

PLOS ONE

Hormonal Contraceptives and Body Composition by Use of Stable Isotope Dilution Techniques Among Women of Reproductive Age in Nyeri County, Kenya

--Manuscript Draft--

**Manuscript Number:**	PONE-D-25-16297
**Article Type:**	Research Article
**Full Title:**	Hormonal Contraceptives and Body Composition by Use of Stable Isotope Dilution Techniques Among Women of Reproductive Age in Nyeri County, Kenya
**Short Title:**	Hormonal Contraceptives and Body Composition of Women of Reproductive Age in Nyeri County, Kenya
**Corresponding Author:**	Purity Lang'at<br>Kenyatta University<br>Nairobi, KENYA
**Keywords:**	Percent Body Fat<br>Total Body Water<br>Body Composition<br>Hormonal Contraceptives<br>Women of Reproductive Age
**Abstract:**	Background: Hormonal contraceptives are widely used by women of reproductive age (WRA) globally, yet their impact on body composition remains poorly understood, particularly in low-resource settings. This study aimed to assess the effects of hormonal contraceptives on body composition among WRA in Nyeri County, Kenya, using Stable Isotope Dilution Techniques (SIDT).<br>Methods: A two-stage cross-sectional analytical study was conducted among 114 women of reproductive age (WRA) aged 18–49 years. Participants were categorized into three groups: hormonal contraceptive users (n=35), non-hormonal contraceptive users (n=36), and non-contraceptive users (n=33). Baseline data collection included demographic, socioeconomic, and reproductive health characteristics (assessed via structured questionnaires), anthropometric measurements, and body composition analysis using SIDT. Participants were followed up monthly via phone calls, with a 3-month onsite assessment to evaluate contraceptive compliance, side effects, and repeat anthropometric/body composition measurements. A final SIDT assessment was conducted at six months. Physical activity and dietary diversity were assessed to control for confounding factors.<br>Results: Hormonal contraceptive users exhibited a significant reduction in total body water (42.3 ± 7.8%) and an increase in body fat percentage (41.7 ± 10.6) compared to non-hormonal users (33.4±10.7) and non-contraceptive users (36.5±7.4) ($p < 0.05$). No significant changes were observed in fat-free mass or fat mass across the groups. Physical activity and dietary diversity remained stable over the study period, suggesting that hormonal contraceptives independently influenced body composition.<br>Conclusion: Hormonal contraceptives are associated with significant changes in body composition, particularly increased body fat percentage, among WRA in Nyeri County. These findings underscore the need for routine body composition monitoring and nutritional guidance for women on hormonal contraceptives to mitigate potential metabolic risks.
**Order of Authors:**	Purity Lang'at, MSc. Foods, Nutrition and Dietetics
	Dorcus Mbithe David-Kigaru, PhD
	Zipporah W. Ndung’u, PhD
	Pamela Kimeto, PhD
**Additional Information:**	
**Question**	**Response**
**Financial Disclosure**	The author(s) received no specific funding for this work.

Enter a financial disclosure statement that describes the sources of funding for the work included in this submission. Review the submission guidelines for detailed requirements. View published research articles from *PLOS ONE* for specific examples.<br><br>This statement is required for submission and **will appear in the published article** if the submission is accepted. Please make sure it is accurate.<br><br>**Funded studies**<br>Enter a statement with the following details:<br>• Initials of the authors who received each award<br>• Grant numbers awarded to each author<br>• The full name of each funder<br>• URL of each funder website<br>• Did the sponsors or funders play any role in the study design, data collection and analysis, decision to publish, or preparation of the manuscript?<br><br>Did you receive funding for this work?	
**Competing Interests**<br><br>Use the instructions below to enter a competing interest statement for this submission. On behalf of all authors, disclose any competing interests that could be perceived to bias this work—acknowledging all financial support and any other relevant financial or non-financial competing interests.<br><br>This statement is required for submission and **will appear in the published article** if the submission is accepted. Please make sure it is accurate and that any funding sources listed in your Funding Information later in the submission form are also declared in your Financial Disclosure statement.<br><br>View published research articles from	The authors have declared that no competing interests exist.

[*PLOS ONE*] for specific examples.<br><br>**NO authors have competing interests**<br><br>Enter: *The authors have declared that no competing interests exist.*<br><br>**Authors with competing interests**<br><br>Enter competing interest details beginning with this statement:<br><br>*I have read the journal's policy and the authors of this manuscript have the following competing interests: [insert competing interests here]*<br><br>* typeset	
**Ethics Statement**<br><br>Enter an ethics statement for this submission. This statement is required if the study involved:<br><br>• Human participants<br>• Human specimens or tissue<br>• Vertebrate animals or cephalopods<br>• Vertebrate embryos or tissues<br>• Field research<br><br>Write "N/A" if the submission does not require an ethics statement.<br><br>General guidance is provided below. Consult the submission guidelines for detailed instructions. **Make sure that all information entered here is included in the Methods section of the manuscript.**	This study obtained ethical clearance from the Kenyatta University Ethical Review Committee number PKU2568/11694.<br><br>These approvals facilitated securing a permit from the National Commission of Science, Technology, and Innovation (NACOSTI) License number NACOSTI/P/22/20045.<br><br>Informed written voluntary consent was obtained from all participants, emphasizing their right to withdraw from the study at any time without repercussions.<br><br>The community was fully informed about the study's objectives, potential benefits, and any foreseeable risks.<br><br>The study adhered rigorously to Ministry of Health and International Atomic Energy Agency (IAEA) guidelines on COVID-19 to ensure participant and researcher safety during data collection.

**Format for specific study types**<br><br>**Human Subject Research (involving human participants and/or tissue)**<br>• Give the name of the institutional review board or ethics committee that approved the study<br>• Include the approval number and/or a statement indicating approval of this research<br>• Indicate the form of consent obtained (written/oral) or the reason that consent was not obtained (e.g. the data were analyzed anonymously)<br><br>**Animal Research (involving vertebrate animals, embryos or tissues)**<br>• Provide the name of the Institutional Animal Care and Use Committee (IACUC) or other relevant ethics board that reviewed the study protocol, and indicate whether they approved this research or granted a formal waiver of ethical approval<br>• Include an approval number if one was obtained<br>• If the study involved *non-human primates*, add *additional details* about animal welfare and steps taken to ameliorate suffering<br>• If anesthesia, euthanasia, or any kind of animal sacrifice is part of the study, include briefly which substances and/or methods were applied<br><br>**Field Research**<br><br>Include the following details if this study involves the collection of plant, animal, or other materials from a natural setting:<br>• Field permit number<br>• Name of the institution or relevant body that granted permission	
**Data Availability**<br><br>Authors are required to make all data underlying the findings described fully available, without restriction, and from the time of publication. PLOS allows rare exceptions to address legal and ethical concerns. See the PLOS Data Policy and FAQ for detailed information.	Yes - all data are fully available without restriction

A Data Availability Statement describing where the data can be found is required at submission. Your answers to this question constitute the Data Availability Statement and **will be published in the article**, if accepted.<br><br>**Important:** Stating 'data available on request from the author' is not sufficient. If your data are only available upon request, select 'No' for the first question and explain your exceptional situation in the text box.<br><br>Do the authors confirm that all data underlying the findings described in their manuscript are fully available without restriction?	
**Describe where the data may be found in full sentences. If you are copying our sample text, replace any instances of XXX with the appropriate details.**<br><br>• If the data are **held or will be held in a public repository**, include URLs, accession numbers or DOIs. If this information will only be available after acceptance, indicate this by ticking the box below. For example: *All XXX files are available from the XXX database (accession number(s) XXX, XXX.)*.<br>• If the data are all contained **within the manuscript and/or Supporting Information files**, enter the following: *All relevant data are within the manuscript and its Supporting Information files.*<br>• If neither of these applies but you are able to provide **details of access elsewhere**, with or without limitations, please do so. For example:<br><br>*Data cannot be shared publicly because of [XXX]. Data are available from the XXX Institutional Data Access / Ethics Committee (contact via XXX) for researchers who meet the criteria for access to confidential data.*<br><br>*The data underlying the results presented in the study are available from (include the name of the third party*	All relevant data are within the manuscript and its Supporting Information files.

and contact information or URL).<br>• This text is appropriate if the data are owned by a third party and authors do not have permission to share the data.<br><br>* typeset	
Additional data availability information:	

Manuscript

Click here to access/download;Manuscript;Hormonal Contraceptives and Body Composition.docx

Hormonal Contraceptives and Body Composition by Use of Stable Isotope Dilution Techniques Among Women of Reproductive Age in Nyeri County, Kenya

Purity Lang'at[1,2], Dorcus Mbithe David-Kigaru [1], Zipporah W. Ndung'u[3], Pamela Kimeto[2]

Institution:

1. Kenyatta University, Department of Food, Nutrition and Dietetics. P.O Box 43844-00100 Nairobi, Kenya.
2. Kabarak University, Department of Human Nutrition and Dietetics. P.O Box Private Bag-20157 Kabarak, Kenya,
3. Jomo Kenyatta University of Agriculture and Technology, Department of Human Nutrition Sciences. P.O Box 62000-00200, Nairobi, Kenya

Corresponding Author: Purity Lang'at lapuritie@gmail.com

ABSTRACT

Background: Hormonal contraceptives are widely used by women of reproductive age (WRA) globally, yet their impact on body composition remains poorly understood, particularly in low-resource settings. This study aimed to assess the effects of hormonal contraceptives on body composition among WRA in Nyeri County, Kenya, using Stable Isotope Dilution Techniques (SIDT).

Methods: A two-stage cross-sectional analytical study was conducted among 114 women of reproductive age (WRA) aged 18–49 years. Participants were categorized into three groups: hormonal contraceptive users (n=35), non-hormonal contraceptive users (n=36), and non-contraceptive users (n=33). Baseline data collection included demographic, socioeconomic, and reproductive health characteristics (assessed via structured questionnaires), anthropometric measurements, and body composition analysis using SIDT. Participants were followed up monthly via phone calls, with a 3-month onsite assessment to evaluate contraceptive compliance, side effects, and repeat anthropometric/body composition measurements. A final SIDT assessment was conducted at six months. Physical activity and dietary diversity were assessed to control for confounding factors.

Results: Hormonal contraceptive users exhibited a significant reduction in total body water (42.3 ± 7.8%) and an increase in body fat percentage (41.7 ± 10.6) compared to non-hormonal users (33.4±10.7) and non-contraceptive users (36.5±7.4) ($p < 0.05$). No significant changes were observed in fat-free mass or fat mass across the groups. Physical activity and dietary diversity remained stable over the study period, suggesting that hormonal contraceptives independently influenced body composition.

Conclusion: Hormonal contraceptives are associated with significant changes in body composition, particularly increased body fat percentage, among WRA in Nyeri County. These findings underscore the need for routine body composition monitoring and nutritional guidance for women on hormonal contraceptives to mitigate potential metabolic risks.

Introduction

Globally, over 1.9 billion women are of reproductive age (WRA), with more than 1.1 billion requiring family planning services (United Nations, 2019). In Sub-Saharan Africa, 28.5% of WRA use modern contraceptives, with Kenya reporting one of the highest rates at 43.8% (2). Hormonal contraceptives, including injectables, pills, and implants, are widely used due to their effectiveness in preventing unintended pregnancies. However, their potential side effects, including changes in body composition, remain a concern (3).

Body composition, which includes fat mass (FM) and fat-free mass (FFM), is a critical indicator of nutritional status and overall health. Hormonal contraceptives, particularly those containing estrogen and progestin, have been linked to changes in body weight, fat distribution, and fluid retention (4). Despite the widespread use of hormonal contraceptives, there is limited research on their impact on body composition, especially in low-resource settings like Kenya.

This study aimed to fill this gap by investigating the effects of hormonal contraceptives on body composition among WRA in Nyeri County, Kenya, using Stable Isotope Dilution Techniques (SIDT). SIDT is a non-invasive, accurate method for assessing body composition, making it suitable for use in resource-limited settings (5). While studies have examined hormonal contraceptives' impact on weight gain, few have utilized SIDT to quantify changes in body composition, particularly in Sub-Saharan Africa.

Methods

Study Design and Population

A two-stage cross-sectional analytical study was conducted among 119 WRA aged 18–49 years in Nyeri County, Kenya. The study was conducted in Kamakwa ward, Nyeri Central Sub-County in Nyeri County. The County has reported high levels of over nutrition and related health risks such as diabetes which are usually associated with obesity and increased body fat percentage (6). Over two thirds of WRA in Nyeri County use the various types of contraceptives (7). The Nyeri Town Health Centre and Nyeri Municipal market were used for data collection due to the ease of access by the participants and access to electricity for the data collection tools. Participants were

purposively sampled and categorized into three groups: hormonal contraceptive users, non-hormonal contraceptive users, and non-contraceptive users. Baseline data were collected, followed by a six-month follow-up to assess changes in body composition. While this study assessed changes in body composition over six months, the design does not establish causality between contraceptive use and body composition shifts.

Data Collection

Body Composition Assessment:

This study utilized Deuterium Oxide ($^{2}H_2O$) to assess body composition. Deuterium Oxide, also known as Deuterium, is a stable isotope of hydrogen. The preparation and dosing of participants followed the standard operating procedures outlined by the International Atomic Energy Agency (IAEA) SOPs protocols (5). This involved the administration of deuterium oxide (D_2O) and the collection of saliva samples before and after dosing. Total body water (TBW), fat-free mass (FFM), and fat mass (FM) were calculated using established formulas (8). Anthropometric measurements, including weight and height, were also recorded.

Questionnaire Administration:

Demographic, socioeconomic, and reproductive health data were collected using structured questionnaires. Physical activity levels were assessed using the Global Physical Activity Questionnaire (GPAQ), and dietary diversity was measured using the Women's Dietary Diversity Score (WDDS).

Sample Size and Sampling:

The sample size was calculated using Fisher's formula, with a 95% confidence level and a 5% margin of error. A total of 119 participants were recruited, with 10% added to account for non-response.

Statistical Analysis

Data were analyzed using SPSS version 25. Descriptive statistics were used to summarize demographic and socioeconomic characteristics. Changes in body composition were analyzed using paired t-tests and ANOVA. Multiple regression analysis was conducted to identify factors influencing body composition, with significance set at $p < 0.05$.

Ethical Considerations

The study obtained ethical clearance from the Kenyatta University Ethical Review Committee and approval from the Kenyatta University Graduate School. Additionally, a research permit was secured from the National Commission for Science, Technology, and Innovation (NACOSTI).

All participants provided written informed consent before participating in the study. They were informed about the study's objectives, procedures, potential risks, and benefits. Participants were assured of their right to withdraw from the study at any time without any consequences. Consent forms were available in both English and Kiswahili to ensure comprehension.

Participant data were treated with strict confidentiality. All personal identifiers were removed, and data were stored securely in password-protected electronic files. Access to the data was restricted to authorized research team members only. Data collection tools were programmed into tablets equipped with the Kobo Collect application, which facilitated secure data transmission to a central server within 24 hours of collection.

The study adhered to Ministry of Health and International Atomic Energy Agency (IAEA) guidelines on COVID-19. Participants and researchers were required to wear masks, maintain social distancing, and use hand sanitizers during data collection. Equipment was sanitized before and after each use to minimize the risk of infection.

Results

Demographic and Socioeconomic Characteristics

The mean age of participants was 26.01 ± 7.46 years, with 46.2% aged 20–29 years. Most participants (50.9%) were married, and 49.1% had primary education. The majority (78.9%) earned less than KSh. 20,000 per month (Table 1).

Table 1: Demographic and Socioeconomic Characteristics of Study Women

Variable	Baseline		Endline		
	N=114	%	N=104	%	χ2 p-value
**Mean age**	26.01±7.46		26.20±7.58		0.967
**Age**					
20-29	53	46.2	49	47.2	
30-39	40	35.1	36	34.6	
40-49	21	18.4	19	18.3	
**Education level**					
Primary	56	49.1	50	48.1	0.986
Secondary	47	41.2	44	42.3	
Tertiary/college/university	11	9.6	10	9.6	
**Occupation**					
Farmer	11	9.6	12	11.5	0.948
Casual labor	53	46.5	44	42.3	
Salaried labor	6	5.3	4	3.8	
Business	34	29.8	33	31.7	
Student	1	0.9	2	1.9	
Housewife	9	7.9	9	8.7	
**Respondent monthly income**					
No income	6	5.3	6	5.5	0.847
≤20,000	90	78.9	81	77.9	
20,001-40,000	16	14.0	15	14.4	
40,001-60,000	1	0.9	2	1.9	
≥60,001	1	0.9	0	0.0	

*Significant at p<0.05

Contraceptive Use

At baseline, 68.4% of participants were using contraceptives, with hormonal methods (36.0%) being the most common. Health workers were the primary source of contraceptive information (71.8%), and government clinics were the main source of contraceptives (84.6%) (Table 2).

Table 2: Types of Contraceptives Used by Study Women

**Characteristic**	**Baseline N=114**	**%**	**Endline N=104**	**%**	**χ2 p-value**
Number of WRA on contraceptives	78	68.4	71	68.3	0.996
**Type of contraceptive used**					
Injectables	12	10.5	10	9.6	0.886
Pills	13	11.4	10	9.6	
Implants	16	14.0	15	14.4	
IUD	22	19.3	22	21.2	
Male/Female condom	15	13.2	14	13.5	
None	36	31.6	33	31.7	
**Categories of contraceptives used**					
Hormonal	41	36.0	35	33.7	0.804
Non-hormonal	37	32.5	36	34.6	
No contraceptive	36	31.5	33	31.7	
**Source of information on choice of contraceptives**					
Self	4	5.1	3	4.2	0.136
Healthworker	56	71.8	57	80.3	
Colleagues	2	2.6	1	1.4	
Spouse	12	15.4	8	11.3	
Media	3	3.8	2	2.8	
Friends	2	2.6	2	2.8	
**Main source of contraceptives**					
Government clinic or hospital	66	84.6	63	88.7	0.933
Pharmacy	4	5.1	4	5.6	
Private hospitals	6	7.6	4	5.6	
General Shop	3	3.8	2	2.8	

*Significant at p<0.05

Body Composition Characteristics

Hormonal contraceptive users experienced a significant increase in body fat percentage (35.3 ± 9.3% to 41.7 ± 10.6%, p = 0.039), alongside a reduction in total body water (47.4 ± 6.8% to 42.3 ± 7.8%, p = 0.048) over six months. This suggests that hormonal contraceptives may promote fluid redistribution and adipose tissue accumulation. In contrast, non-hormonal users and non-users showed no significant changes in body composition (p > 0.05). Notably, hormonal users' fat mass index (FMI) trended upward (10.0 ± 4.8 to 12.4 ± 5.1, p = 0.359), though not statistically significant, further supporting a potential shift toward adiposity. (Table 3).

Table 3: Changes in the Mean Body Composition Characteristics of Women of Reproductive Age

	**Hormonal (n=35)**			**Non- Hormonal (n=36)**			**Not on Contraceptives (n=33)**		
	**Baseline**	**Endline**	**Paired t-test**	**Baseline**	**Endline**	**Paired t-test**	**Baseline**	**Endline**	**Paired t-test**
Total Body Water (Kg)	32.0±5.4	29.7±5.5	**0.040***	32.5±6.9	32.6±6.1	0.858	32.0±5.6	31.9±4.7	0.662
Fat-Free Mass (Kg)	40.2±7.4	36.0±7.6	**0.038***	44.4±9.4	44.6±9.4	0.845	43.7±7.6	43.6±6.4	0.854
Fat Mass (Kg)	26.0±12.8	29.0±13.7	0.165	25.9±11.6	24.3±14.3	0.870	25.6±9.5	26.0±9.1	0.867
Fat-Free Mass Index	16.9±2.7	14.3±2.8	0.068	17.2±3.1	17.3±3.9	0.855	16.7±2.7	16.7±2.5	0.760
Fat Mass Index	10.0±4.8	12.4±5.1	0.359	10.1±4.0	9.2±5.1	0.562	9.8±3.4	9.9±3.5	0.898
Total Body Water (%)	47.4±6.8	42.3±7.8	**0.048***	47.0±5.2	48.7±7.9	0.665	46.8±6.4	46.5±5.4	0.896
Body Fat (%)	35.3±9.3	41.7±10.6	**0.039***	35.8±7.2	33.4±10.7	0.460	36.0±8.7	36.5±7.4	0.885

*Significance at p<0.05

Respondents with Excess Body Fat Percent at Baseline and Endline by the Type of Contraceptive Used

The study further sought to analyze changes in body fat percentage among the study participants from baseline to endline. The proportion of participants with excess body fat increased from

40.4% at baseline to 47.1% at endline. However, this change was not statistically significant (p=0.078) (Table 4).

At baseline, the proportion of participants with normal fat mass was relatively similar across the three groups: 57.1% for hormonal, 61.1% for non-hormonal, and 60.6% for no contraceptive use. There was no significant difference in body fat percentage among participants based on contraceptive use at baseline (p=0.094) (Table 4).

At endline, 60.0% of participants using hormonal contraceptives had excess fat mass, compared to 41.6% for those on non-hormonal contraceptives. Among those not using contraceptives, the proportion with excess fat mass remained unchanged at 39.4%. A significant change in body fat percentage was observed among those using hormonal contraceptives (p=0.034), while there was no significant change among those using non-hormonal contraceptives (p=0.086) or those not on contraceptives (p=0.983). These changes persisted despite stable physical activity and dietary diversity scores (Table 4).

Table 4: Comparison of Percent Body Fat by the Type of Contraceptive Used at Baseline and Endline

	**Total N (%)**			**Hormonal**			**Non-hormonal**			**Not on contraceptives**		
**Variable**	**Baseline**	**Endline**	**χ2 p-value**	**Baseline n (%) n=35**	**Endline n (%) n=35**	**χ2 p-value**	**Baseline n (%) n=36**	**Endline n (%) n=36**	**χ2 p-value**	**Baseline n (%) n=33**	**Endline n (%) n=33**	**χ2 p-value**
**% Body Fat**												
Normal	62(59.6)	55(52.9)	0.094	20(57.1)	14(40.0)	**0.045***	22(61.1)	21(56.4)	0.079	20(60.6)	20(60.6)	0.095
Excess	42(40.4)	49(47.1)	0.078	15(42.9)	21(60.0)	**0.034***	14(38.9)	15(41.6)	0.086	13(39.4)	13(39.4)	0.983

*Significant at p<0.05

Physical Activity and Dietary Diversity

Physical activity levels and dietary diversity scores remained stable over the study period, indicating that these factors did not confound the observed changes in body composition (Table 5a and b). The stability of dietary patterns suggests that observed body composition changes were more likely attributable to contraceptive use rather than dietary modifications.

Table 5a: Analysis of Physical Activity among Study Women

Physical Activity Level	Baseline N (%) N=114	Endline N (%) N=104	Chi-Square p-value
**Work-Related Activity**			
Vigorous-intensity (Met Value=8)	15 (13.2%)	15 (14.4%)	0.932
Moderate-intensity (Met Value=4)	57 (50.2%)	51 (49.5%)	0.764
**Mean MET**	5.6 ± 1.02	5.5 ± 1.08	0.312
**Travel-Related Activity**			
Walking or cycling for ≥10 min continuously	42 (36.6%)	38 (36.1%)	0.872
**Mean MET**	4.2 ± 0.75	4.1 ± 0.81	0.289
**Recreational Activity**			
Vigorous-intensity (Met Value=8)	15 (13.2%)	14 (13.0%)	0.876
Moderate-intensity (Met Value=4)	57 (50.2%)	52 (50.0%)	0.845
**Mean MET**	5.0 ± 0.95	4.8 ± 1.02	0.246
**Sedentary Behavior**			
Time spent sitting on a typical day (Met Value=4)	72 (63.2%)	68 (60.5%)	0.734
**Mean MET**	1.2 ± 0.25	1.1 ± 0.22	0.212
**Physical Activity Levels**			
Low (MET Value <4)	15 (13.2%)	15 (14.4%)	0.932
Moderate (Met Value ≥4 to < 8)	57 (50.2%)	51 (49.5%)	0.764
High (Met Value = 8)	42 (36.6%)	38 (36.1%)	0.872
**Mean MET**	3.5 ± 0.59	3.4 ± 0.64	0.312

*Significant at p<0.05

Table 5b: Analysis of Women's Dietary Diversity Score

Variable	Baseline		Endline		Chi-Square p-value
	N=114	%	N=104	%	
**Household dietary diversity score**					
<4 food groups (Low)	21	18.8	24	23.1	0.416
≥4 Food groups (Adequate)	93	81.2	80	76.9	
**Women Dietary Diversity Score**					
<5 food groups (Low)	99	87.1	87	83.7	0.385
≥5 food groups (Adequate)	15	12.9	17	16.3	

*Significant at p<0.05

Discussion

Demographic and Socioeconomic Characteristics of the Women of Reproductive Age in Nyeri County, Kenya

The mean age of the women in this study was 26.01±7.46 years, with the majority (46.2%) aged between 20–29 years, followed by those aged 30–39 years (35.1%). The age distribution aligns with findings from other studies in sub-Saharan Africa, where women in their 20s and 30s constitute the largest proportion of women of reproductive age (WRA) due to the typical childbearing years in these regions (7,8). This contrasts with trends observed in more urbanized settings, where delayed childbearing is increasingly linked to longer educational attainment and career aspirations (9,10). The relatively young age of the participants also highlights the importance of targeting reproductive health interventions, including family planning and maternal health services, to younger women who are at the peak of their reproductive years.

The occupational distribution of the participants reflects the predominantly informal economy in rural Kenya, with 46.5% engaged in casual labor and 29.8% in business. Smaller proportions were involved in farming (9.6%), salaried employment (5.3%), and housework (7.9%). Casual labor, characterized by low wages, job insecurity, and lack of benefits, is a common occupation among low-income women in rural areas (13). The high prevalence of women in informal business activities indicates entrepreneurial resilience, although the scale and profitability of these ventures are likely constrained by limited access to capital, markets, and business skills. The small percentage of salaried labor reflects the limited formal employment opportunities available in rural settings, further contributing to economic vulnerability. Notably, only 0.9% of the respondents were students, highlighting a gap in adult education or vocational training opportunities for women. This gap represents a missed opportunity to enhance the socioeconomic status of WRA through education, which could empower them with better employment prospects and access to health services (14).

Income levels among the participants were low, with 78.9% earning less than KSh. 20,000 per month and only 1% earning more than KSh. 60,000. This income distribution mirrors findings from other studies in Kenya, where low household incomes among women in rural areas are often linked to high levels of poverty and economic insecurity (13,14).

Reproductive Health and Contraceptive Use

The study shows that that 68.3% of the WRA were using some form of contraception, a figure that compares favourably with national trends in Kenya, where the modern contraceptive prevalence rate (mCPR) among married women is around 58% (17). The types of contraceptives used, including injectables (10.5%), pills (11.4%), implants (14.0%), intrauterine devices (IUDs) (19.3%), and condoms (13.2%), reflect the range of options available to women in public health facilities. The relatively high use of IUDs and implants, which are long-acting reversible contraceptives (LARCs), is consistent with other studies indicating a growing preference for these methods among women seeking longer-term protection from pregnancy (16,17,18).

The study's finding that 31.6% of WRA were not using any contraceptive method underscores a persistent gap in contraceptive uptake. This non-use could stem from several factors, including fear of side effects, lack of access, religious or cultural beliefs, or misinformation about contraceptive methods. For example, qualitative studies from Kenya (19,20) and other parts of Africa (23) have shown that concerns about side effects, such as weight gain, irregular menstruation, or perceived long-term health risks, are major reasons for non-use of contraception.

The finding that health workers were the primary source of information (70.9%) influencing contraceptive choice is consistent with other studies showing that healthcare providers play a crucial role in promoting family planning services. Government clinics and hospitals were also the predominant source of contraceptives for most participants (83.5%), underscoring the role of public health systems in contraceptive provision. This is a significant advantage, especially for low-income women who may not be able to afford contraceptives from private providers.

Body Composition of Women of Reproductive Using Stable Isotope Dilution Techniques

Hormonal contraceptive users exhibited a significant reduction in percent TBW (42.3 ± 7.8) comparing baseline and endline TBW (p= 0.048) while the non-hormonal and non-contraceptive users, there were no significant changes in % TBW (p=0.665, p=0.896) respectively. This reduction in TBW may be attributed to the fluid-retentive effects of hormonal contraceptives, particularly those containing estrogen and progestin. Estrogen is known to influence sodium and water retention by modulating renal function and aldosterone activity, which can lead to changes in fluid distribution and electrolyte balance (24). Similarly, progestins in hormonal contraceptives can affect fluid balance by altering the renin-angiotensin-aldosterone system (RAAS), leading to increased water retention in tissues (4). These findings align with previous studies that have reported similar reductions in TBW among women using combined oral contraceptives (COCs) and depot medroxyprogesterone acetate (DMPA) (25).

Hormonal contraceptive users had a significantly higher body fat percentage (41.7 ± 10.6) at endline compared to non-hormonal users (33.4 ± 10.7%) and non-contraceptive users (36.5 ± 7.4) (p = 0.039). This increase in body fat is consistent with the known effects of hormonal contraceptives on fat metabolism and storage. Progestins, in particular, have been shown to promote adipogenesis (fat cell formation) and inhibit lipolysis (fat breakdown), leading to increased fat deposition (26). Additionally, hormonal contraceptives can influence appetite and energy expenditure, further contributing to weight gain and fat accumulation (27). This aligns with previous research suggesting that hormonal contraceptives, particularly progestin-based methods, may influence fat deposition and metabolic processes (25,26). The minimal changes observed in non-hormonal users and those not using contraceptives further support the hypothesis that hormonal contraceptives play a direct role in altering body composition. These findings are consistent with studies that have reported weight gain and increased adiposity as common side effects of hormonal contraceptives (3).

While there were no significant changes in fat mass (FM) or fat-free mass (FFM) across the total sample, hormonal contraceptive users showed a trend toward higher FM and lower FFM at endline. This trend is consistent with the observed increase in body fat percentage and suggests that hormonal contraceptives may preferentially affect fat deposition rather than lean mass. The

analysis of body fat percentage changes among WRA based on contraceptive use provides further evidence of the impact of hormonal contraceptives on body composition. At baseline, the proportion of participants with normal body fat percentage was similar across all contraceptive groups (57.1% for hormonal, 61.1% for non-hormonal, and 60.6% for non-contraceptive users). However, by endline, hormonal contraceptive users had a significantly higher proportion of excess body fat (60.0%) compared to non-hormonal users (41.6%) and non-contraceptive users (39.4%) ($p = 0.034$). This increase in excess body fat among hormonal users is consistent with previous studies that have reported weight gain and fat accumulation as common side effects of hormonal contraceptives (3,4).

The observed increase in excess body fat among hormonal contraceptive users may have important implications for women's health. Excess body fat, particularly central adiposity, is a known risk factor for metabolic disorders such as type 2 diabetes, cardiovascular disease, and hypertension (29). While the current study did not assess the long-term health outcomes associated with these changes, the findings suggest that hormonal contraceptive use may contribute to an increased risk of metabolic complications, particularly among women who are already overweight or obese.

Analysis of Physical activity and Dietary Diversity Score

Physical activity levels among women remained largely unchanged between baseline and endline across all domains, including work-related, travel-related, recreational activities, and sedentary behaviour. The physical activity data in this study shows that most WRA in Nyeri County lead moderately active lifestyles, with low levels of vigorous and moderate activity. This is consistent with findings from other studies in sub-Saharan Africa, which have shown that physical activity levels among women are often low due to a combination of cultural, social, and economic factors. For example, Assah et. al., (28) found that women in urban Cameroon had low levels of physical activity, which was associated with higher rates of obesity and related metabolic disorders. Similarly, Muthuri et. al., (29) reported that women in rural Kenya engaged in low

levels of moderate-to-vigorous physical activity, which was associated with higher body fat percentages.

In this study, the stability of physical activity levels between baseline and endline suggests that physical activity did not significantly change over the study period. This is important because changes in physical activity could confound the relationship between hormonal contraceptives and body composition. By controlling for physical activity, we can more confidently attribute any observed changes in body composition to the influence of hormonal contraceptives.

The dietary diversity scores remained relatively stable between baseline and endline. At baseline, 81.2% of households consumed more than 4 food groups, indicating relatively good dietary diversity. Only 12.9% of women at baseline and 15.9% at endline consumed 5 or more food groups, which is the minimum recommended for adequate micronutrient intake. The majority (87.1% at baseline and 84.1% at endline) consumed fewer than 5 food groups, indicating potential micronutrient deficiencies.

In this study, the stability of dietary diversity scores between baseline and endline suggests that dietary habits did not significantly change over the study period. This is important because changes in dietary intake could confound the relationship between hormonal contraceptives and nutritional status. By controlling for dietary diversity, we can more confidently attribute any observed changes in nutritional status to the influence of hormonal contraceptives.

The influence of hormonal contraceptives on body composition and nutritional status has been widely studied, with mixed findings. Some studies have reported that hormonal contraceptives, particularly progestin-only methods, are associated with weight gain and changes in body composition. For example, Gallo et al.,(3) found that women using depot medroxyprogesterone acetate (DMPA) experienced significant increases in body weight and fat mass over a 12-month period. Similarly, Bahamondes et al., (32) reported that women using DMPA had higher rates of weight gain and increased body fat compared to non-contraceptive users.

However, other studies have found no significant association between hormonal contraceptives and changes in body composition. For example, Lopez et al., (28) found no significant

differences in body weight or fat mass between women using combined oral contraceptives and non-contraceptive users over a 24-month period. Similarly, (33) reported that women using DMPA did not experience significant changes in body composition compared to non-contraceptive users.

This study contributes to the ongoing debate on hormonal contraceptives and body composition by demonstrating significant changes in adiposity and hydration among users—independent of dietary diversity and physical activity, which were rigorously controlled. By accounting for these key confounders, the findings strengthen the evidence that hormonal contraceptives may directly influence body fat percentage and fluid balance, rather than such changes being driven by lifestyle factors. These results underscore the need to consider contraceptive type in nutritional and metabolic health assessments for women of reproductive age.

Delimitation of the Study

This study focussed on examining effects of hormonal contraceptives on the body composition and nutritional status of women of reproductive age residing in Kamakwa Ward of Nyeri Central Sub-County, Nyeri County Kenya. Findings can therefore be generalized only to populations of similar characteristics as the ward in this study.

Limitation of the Study

The study excluded the women who were either pregnant or lactating with children less than 1 year of age due to the accumulation of body fat related to these physiological phases.

To address the potential presence of confounding variables such as diet, exercise habits, socioeconomic status, and underlying health conditions that could influence the relationship between contraceptives and body composition, efforts were made to collect comprehensive data on participants' lifestyle factors and health status to facilitate more accurate adjustments for potential confounders in the analysis.

As a cross-sectional study with a six-month follow-up, causal relationships could not be established. Further, the study did not differentiate between specific types of hormonal contraceptives (e.g., oral pills, injectables, implants), which may have varying effects on body composition and nutritional status. This limits the ability to conclude the impact of specific contraceptive formulations.

Conclusions

This study revealed that hormonal contraceptive use was associated with significant increases in body fat percentage and reductions in total body water, while no such changes were observed among non-hormonal users or non-contraceptive users. These findings suggest that hormonal contraceptives may uniquely influence body composition dynamics, potentially promoting adiposity and fluid redistribution over time. By controlling for dietary diversity and physical activity, the study strengthens the evidence for a direct biological effect of hormonal contraceptives on fat accumulation, underscoring their role as a modifiable factor in women's metabolic health.

Recommendations

Monitoring of body composition changes, particularly body fat percentage, among women using hormonal contraceptives by incorporating body composition assessments into primary healthcare visits, to track and manage potential adverse effects.

Providing nutritional guidance and support to women using hormonal contraceptives to mitigate potential increases in body fat percentage and promote overall health by developing and disseminating educational materials on healthy eating and physical activity tailored to women using hormonal contraceptives. Collaborate with nutritionists to offer personalized dietary advice.

Acknowledgements

I extend my sincere appreciation to the UN International Atomic Energy Agency (IAEA), Vienna for funding this work through regional project KEN 6025, within which this study was nested. I am grateful to the County Government of Nyeri for granting permission to conduct this

study in their institution and to the administration of Kabarak University for allowing me to pursue my studies.

I deeply appreciate Dr. Peter Chege for his invaluable support in data analysis.

My heartfelt gratitude also goes to the staff at Nyeri Town Health Centre and the women of reproductive age who participated in this study—without their involvement, this research would not have been possible.

Author Contribution

Conceptualization: Purity Lang'at, Dorcus Mbithe

Research Methodology: Dorcus Mbithe, Zipporah Ndung'u, Pamela Kimeto, Purity Langat

Data Collection, Analysis and Presentation: Dorcus Mbithe, Zipporah Ndung'u, Purity Langat

Resources: Dorcus Mbithe

Writing, Review and Editing: Dorcus Mbithe, Zipporah Ndung'u, Pamela Kimeto, Purity Langat

Conflicting interests

The authors declare that they have no conflicting interest.

REFERENCES

1. United Nations, Department of Economic and Social Affairs PD. Family Planning and the 2030 Agenda for Sustainable Development: Data Booklet. Fam Plan 2030 Agenda Sustain Dev (Data Booklet) [Internet]. 2019; Available from: https://www.un.org/en/development/desa/population/publications/pdf/family/familyPlanning_DataBooklet_2019.pdf

2. WHO. World Health Organization Bulletin. 2020.

3. Gallo MF, Lopez LM, Grimes DA, Carayon F, Schulz KF, Helmerhorst FM. Combination contraceptives: effects on weight. Cochrane database Syst Rev. 2014 Jan;2014(1):CD003987.

4. Abbey B. B, Rahman M. Changes in weight, total fat, percent body fat, and central-to-peripheral fat ratio associated with injectable and oral contraceptive use. Am J Obs Gynecol. 2009;23(1):1–7.

5. IAEA IAEA. Introduction to Body Composition Assessment using the Deuterium Dilution Technique with Analysis of Urine Samples by Isotope Ratio Mass Spectrometry. [Internet]. IAEA Human Health Series no. 13. 2010. Available from: http://doi.org/http://www-pub.iaea.org/MTCD/publications/PDF/Pub1450_web.pdf

6. Kahanya K. Impact of Maternal Dietary Intake On Breast Milk Composition And Infant Nutrition Status Among Lactating Women In Nyeri County, Kenya. Kenyatta University; 2020.

7. Ndiritu M, Anyango R, Ombech E, Mwita CC, Gwer S. Family Planning Services in Kenya during a Transition: Utilization Trends across Counties. 2017;(April). Available from: www.measureevaluation.org

8. Wells JCK, Fewtrell MS. Measuring body composition. Arch Dis Child [Internet]. 2006 Jul 1;91(7):612 LP – 617. Available from: http://adc.bmj.com/content/91/7/612.abstract

9. Ameyaw EK, Budu E, Sambah F, Baatiema L, Appiah F, Seidu A-A, et al. Prevalence and determinants of unintended pregnancy in sub-Saharan Africa: A multi-country analysis of demographic and health surveys. PLoS One [Internet]. 2019;14(8):e0220970. Available from: http://10.0.5.91/journal.pone.0220970

10. Shasha L, Phiri M, Namayawa S, Sikaluzwe M, Nakazwe C, Lemba M, et al. Prevalence and factors associated with early childbearing in sub-saharan Africa: evidence from demographic and health surveys of 31 countries. BMC Womens Health [Internet]. 2023;23(1). Available from: http://10.0.4.162/s12905-023-02581-z

11. Barreca A, Deschenes O, Guldi M. Maybe Next Month? Temperature Shocks and Dynamic Adjustments in Birth Rates. Demography. 2018;55(4):1269–93.

12. Sobotka T. POST-TRANSITIONAL FERTILITY: THE ROLE OF CHILDBEARING POSTPONEMENT IN FUELLING THE SHIFT TO LOW AND UNSTABLE FERTILITY LEVELS. J Biosoc Sci. 2017 Nov;49(S1):S20–45.

13. Floro MS, Swain RB. Food security, gender, and occupational choice among urban low-income households. World Dev. 2013;(42):89–99.

14. Wekwete NN. Gender and economic empowerment in Africa: Evidence and policy. J Afr Econ. 2014;23((suppl_1)):i87–127.

15. Kroeker L, Boanada-Fuchs A, Gez YN, Waldmüller JM. The Kenyan middle class and responses to social security: Searching Nairobi‘s Middle Class, Kompreno. 2016.

16. Suri T, Tschirley DL, Irungu C, Gitau R, Kariuki D. Rural incomes, inequality and poverty dynamics in Kenya. 2008.

17. Moon TD, Okoth V, Starnes JR, Opiyo E, Ressler DJ, Mbeya J, et al. Determinants of modern contraceptive prevalence and unplanned pregnancies in Migori County, Kenya: results of a cross-sectional household survey. Afr J Reprod Health. 2021 Feb;25(1):29–40.

18. Kavanaugh ML, Jerman J, Finer LB. Changes in Use of Long-Acting Reversible Contraceptive Methods Among U.S. Women, 2009-2012. Obstet Gynecol. 2015 Nov;126(5):917–27.

19. Ontiri S, Ndirangu G, Kabue M, Biesma R, Stekelenburg J, Ouma C. Long-Acting Reversible Contraception Uptake and Associated Factors among Women of Reproductive Age in Rural Kenya. Int J Environ Res Public Health. 2019 May;16(9).

20. Bolarinwa OA, Afaya A, Ajayi K V, Ojo A, Alawode OA. Prevalence and factors associated with the use of long-acting reversible and permanent contraceptive methods among women who desire no more children in high fertility countries in sub-saharan Africa. BMC Public Health [Internet]. 2022;22(1):2141. Available from: https://doi.org/10.1186/s12889-022-14575-x

21. Ochako R, Mbondo M, Aloo S, Kaimenyi S, Thompson R, Temmerman M, et al. Barriers to modern contraceptive methods uptake among young women in Kenya: a qualitative study. BMC Public Health. 2015 Feb;15:118.

22. Ormel H, Oele G, Kok M, Oruko H, Oluoch B, Smet E, et al. Reducing unmet need for contraceptive services among youth in Homabay and Narok counties, Kenya: the role of community health volunteers – a qualitative study. BMC Health Serv Res [Internet]. 2021;21(1):405. Available from: https://doi.org/10.1186/s12913-021-06363-x

23. Engelbert Bain L, Amu H, Enowbeyang Tarkang E. Barriers and motivators of contraceptive use among young people in Sub-Saharan Africa: A systematic review of qualitative studies. PLoS One. 2021;16(6):e0252745.

24. Graugaard-Jensen C, Hvistendahl GM, Frøkiær J, Bie P, Djurhuus JC. Oral Contraceptives and Renal Water Handling: A diurnal study in young women. Physiol Rep. 2017 Dec;5(23).

25. Lopez LM, Ramesh S, Chen M, Edelman A, Otterness C, Trussell J, et al. Progestin-only contraceptives: effects on weight. Cochrane database Syst Rev. 2016

Aug;2016(8):CD008815.

26. Caldwell AE, Zaman A, Ostendorf DM, Pan Z, Swanson BB, Phelan S, et al. Impact of Combined Hormonal Contraceptive Use on Weight Loss: A Secondary Analysis of a Behavioral Weight-Loss Trial. Obesity. 2020;28(6):1040–9.

27. Trussell J, Ministry of Public Health and Sanitation, World Health Organization (WHO), International Atomic Energy Agency I, Silva DRP, Ribeiro AS, et al. Changes in weight, total fat, percent body fat, and central-to-peripheral fat ratio associated with injectable and oral contraceptive use. BMC Public Health [Internet]. 2018 Jul 1 [cited 2022 Feb 9];23(1):1–7. Available from: http://adc.bmj.com/content/91/7/612.abstract

28. Lopez LM, Grimes DA, Chen M, Otterness C, Westhoff C, Edelman A, et al. Hormonal contraceptives for contraception in overweight or obese women. Cochrane database Syst Rev. 2013 Apr;(4):CD008452.

29. Després J-P. Body fat distribution and risk of cardiovascular disease: an update. Circulation. 2012 Sep;126(10):1301–13.

30. Assah FK, Ekelund U, Brage S, Mbanya JC, Wareham NJ. Urbanization, physical activity, and metabolic health in sub-Saharan Africa. Diabetes Care. 2011 Feb;34(2):491–6.

31. Muthuri SK, Wachira L-JM, Leblanc AG, Francis CE, Sampson M, Onywera VO, et al. Temporal trends and correlates of physical activity, sedentary behaviour, and physical fitness among school-aged children in Sub-Saharan Africa: a systematic review. Int J Environ Res Public Health. 2014 Mar;11(3):3327–59.

32. Bahamondes L, Valeria Bahamondes M, Shulman LP. Non-contraceptive benefits of hormonal and intrauterine reversible contraceptive methods. Hum Reprod Update. 2015;21(5):640–51.

33. Berenson AB, Rahman M. Changes in weight, total fat, percent body fat, and central-to-

peripheral fat ratio associated with injectable and oral contraceptive use. Am J Obstet Gynecol [Internet]. 2009;200(3):329.e1-329.e8. Available from: https://www.sciencedirect.com/science/article/pii/S0002937808024605
