# Supplementary material for: Hormonal contraceptives and body composition by use of stable isotope dilution techniques among women of reproductive age in Nyeri County, Kenya
Source: PLoS One. 2025 Sep 12;20(9):e0332299. doi: 10.1371/journal.pone.0332299 (PMC12431291; doi:10.1371/journal.pone.0332299)
Supplement: Attachment — Submitted filename: Respose to reviewers.docx [file pone.0332299.s002.docx]

**RESPONSES TO REVIEWER’S COMMENTS**

**Manuscript Title:** Hormonal Contraceptives and Body Composition by Use of Stable Isotope Dilution Techniques Among Women of Reproductive Age in Nyeri County, Kenya

We thank the editors and reviewers for their thorough and constructive comments. Below is our point-by-point response to each comment, with explanations of how the manuscript was revised. All page numbers refer to the revised manuscript.

**Reviewer #1:**

**Comment:** Grouping all hormonal and non-hormonal contraceptives into broad categories may obscure differences. Specify and justify grouping.

**Response:** Thank you for the observation. We have now clearly listed the specific types of hormonal (DMPA [injectables], oral pills, implants) and non-hormonal (copper IUDs, condoms) contraceptives used in our Methods section (Page 2 and 5). Justification for grouping is also provided due to sample size limitations and common mechanisms of action. This is also acknowledged in the Limitations section (Page 20).

**Reviewer #2:**

**Comment:** Confirm that the work has not been submitted or published elsewhere.

**Response:** We confirm that this manuscript has not been submitted or published elsewhere.

**Reviewer #3:**

**Comment:** Clarify inconsistencies in sample size (114 vs 119 vs 104); also, abstract mentions 3-month follow-up but methods describe 6 months.

**Response:** This has been corrected. The abstract, methods, and results sections now consistently state that 119 participants were recruited, 114 completed baseline SIDT, and 104 completed the endline at six months (Page 2 and 4). A 3-month mid-point check was conducted for compliance only; this has been clarified (Page 5).

**Comment:** Expand Introduction; provide prevalence data, rationale for setting, links to NCDs.

**Response:** We have expanded the Introduction to include contraceptive use prevalence globally and in Kenya, described types of non-hormonal methods, and linked contraceptive use to obesity and NCDs. We have also justified the selection of Kamakwa Ward based on urbanisation trends and NCD burden (Page 5).

**Comment:** Clarify use and validation of data collection tools.

**Response:** The Methods (Page 5) section has been updated to include: Use of standardized tools such as the Global Physical Activity Questionnaire (GPAQ) and the Women’s Dietary Diversity Score (WDDS). Details on pilot testing for cultural relevance and translation validation in the local setting (Page 5).

**Comment:** Include results of multivariate regression analysis and consider visual summary.

**Response:** We have now included the results of a multivariable logistic regression analysis that assessed the association between contraceptive use, physical activity level, dietary diversity score, and increased body fat percentage among women of reproductive age in Nyeri County, Kenya. As presented in Table 6 (Page 14)

**Comment:** Discussion repeats results; compare findings with existing literature.

**Response:** The Discussion (Pages 15–18) has been restructured to reduce repetition. Comparative analysis has been included using: Studies from Ethiopia (Mengesha et al., 2020), United States (Berenson et al., 2009), Uganda and South Africa (NAMSAL trial data, 2021), showing similar patterns in weight gain and body composition among hormonal contraceptive users.

**Comment:** Address referencing inconsistencies.

**Response:** Referencing format has been revised throughout the document for consistency and correct citation order (Page 22 to 24).

**Reviewer #4:**

**Comment:** Minor language, grammar, and formatting issues.

**Response:** All noted typos and formatting issues have been corrected, and the manuscript has been revised for consistent use of British English.

We believe these changes have significantly strengthened the manuscript and are grateful for the opportunity to revise and resubmit.

Sincerely,

***Signed***

**Purity Lang’at**
On behalf of all authors
